# In Vitro Biocontrol Evaluation of Some Selected *Trichoderma* Strains against the Root Pathogen *Fusarium oxysporum* of Hot Pepper (*Capsicum annum* L.) in Bure Woreda, Ethiopia

**DOI:** 10.1155/2022/1664116

**Published:** 2022-07-16

**Authors:** Abayeneh Girma

**Affiliations:** Department of Biology, College of Natural and Computational Science, Mekdela Amba University, P.O. Box 32, Tuluawlia, Ethiopia

## Abstract

*Capsicum annum* L. is a major vegetable crop consumed worldwide as a spice, vegetable, pickle, condiment, and sauce. Each year in Ethiopia, 80% of the yield loss of hot peppers is caused by *Fusarium* wilt. Controlling this disease using fungicides can pollute the environment and induce genetic resistance in fungal phytopathogens. To solve this serious problem, it is necessary to look for economically safe, long-lasting, and effective biocontrol alternatives. Therefore, the objective of this work was to evaluate antagonistically active *Trichoderma* strains against *F*. *oxysporumf.sp.capsici* using a dual culture assay. The results of this study revealed that out of 32 *Trichoderma* isolates tested *in vitro*, only six (*T*. *harzianum* (TD1), *T*. *asperellum* (TD5), *T*. *viride* (TD7), *T*. *hamatum* (TD11), *T*. *virens* (TD15), and *T*. *longibrachiatum* (TD21)) strains showed a varying rate (45.72–93.57%) of biocontrol activity toward the tested pathogen. Of six isolates, three (TD5, TD1, and TD7) strains showed effective antagonists against the root pathogen *Fusarium oxysporum f.sp.capsici* of hot pepper (*C*. *annum*) with a colonization percentage of 89.45%, 90.12%, and 93.57%, respectively. These *Trichoderma* strains showed higher inhibition efficiency (> 70%) against the mycelial growth of *F*. *oxysporum* with good stress tolerance (temperature, pH, salt concentration, and heavy metals) ability. The isolates also produce different hydrolytic enzymes (amylase, protease, cellulase, and chitinase) with mycoparasitism potential against the mycelia growth of *F*. *oxysporum*. Therefore, the results of this study concluded that TD1, TD5, and TD7 *Trichoderma* strains showed potential biocontrol effects with wide stress tolerance ability against the root pathogen *F*. *oxysporum* of hot pepper and can thus be recommended as the best alternative for wide greenhouse and field trial evaluations.

## 1. Introduction

*Capsicum annuum* L. is a vegetable crop, commonly known as bell pepper, sweet pepper, hot pepper, or chili, that is widely grown in several parts of the world. It is consumed as a spice, vegetable, pickle, condiment, and sauce. Internationally, pepper is consumed as a spice and has become an ingredient in medicines and beverages [1]. Hot peppers have high nutritional value, comprising diverse biochemical compounds such as antioxidant phenolic compounds, volatile oils, fatty oils, capsaicinoids, carotenoids, vitamins (A, C, and E), potassium, folic acid, protein, fiber, and mineral elements [2]. However, the heavy loss of hot pepper yield is increasing due to phytopathogens.

Plant disease needs to be controlled to maintain the quality and enhance the productivity of the crops produced by farmers across the world. Beyond good agronomic and agricultural practices, farmers use different approaches to prevent, mitigate, or control plant diseases such as chemical fertilizers, herbicides, fungicides, and pesticides. Such inputs significantly contribute to crop quality and productivity enhancement. However, the environmental pollution caused by excessive use or misuse of agrochemicals is not underlined by most farmers. In addition to causing pollution and harmful effects to human health and the environment, the use of chemicals for the control of plant pathogens also enhances the development of chemical (e.g., fungicide)-resistant phytopathogenic strains [3].

Worldwide, plant fungal diseases are the most important issues in agriculture and food production. It is estimated that losses due to plant diseases account for approximately half of all crop losses in developing countries, with fungal diseases accounting for one-third of these losses. *F*. *oxysporum* is one of the fungal predominant pathogens that induce destructive wilt in more than 100 plants and is ranked 5^th^ out of the 10 most lethal (death-causing) plant pathogens. In Ethiopia, *Fusarium* wilt is the most economically important disease of hot pepper caused by *F*. *oxysporum*. It is responsible for up to 80% of hot pepper yield losses. Recent efforts have focused on developing economically safe, long-lasting, and effective biocontrol methods for the management of plant diseases [4].

Biological control is the suppression of disease by the application of a biocontrol agent (BCA), usually a fungus, bacterium, virus, or a mixture of these, to the plant or the soil. The main advantage of using BCAs is that they are highly specific for a pathogen and hence are considered harmless to nontarget species. Over the past decades, there have been a number of reports conducted on the identification and utilization of effective BCAs for fungal and bacterial diseases in crops, and a number of BCAs are in commercial production. Recently, the use of BCAs has attracted a lot of interest due to the ability of some species to suppress different plant diseases and the possibility of combining them with other control methods. There has also been a great demand for safer, alternative, and effective control agents [1–5].

Globally, among the fungi that constitute effective BCAs, species of the genus *Trichoderma* are well known as model organisms because of their ability to multiply, spread, isolate, and culture easily [6]. The use of *Trichoderma* for biocontrol of *Fusarium* wilt is not only safe for farmers and consumers but also an environmentally acceptable alternative. *Trichoderma* uses several biocontrol mechanisms against the growth of a number of phytopathogenic organisms, including bacteria, nematodes, and purposively fungi (*Pythium*, *Phytophthora*, *Botrytis*, *Rhizoctonia*, and *Fusarium*), by either direct interaction (e.g., hyperparasitism, competition for nutrients and space, and antibiosis) or indirectly by enhancing their ability to take up nutrients, increasing stress tolerance, promoting plant growth, bioremediation of the contaminated rhizosphere, and producing several secondary metabolites, enzymes, and pathogenesis-related (PR) proteins [7–9].

*Trichoderma* has become one of the most researched subjects today, and many commercial preparations have been developed and used against plant pathogens [6]. In Ethiopia, many studies have been conducted on the biocontrol of *F*. *oxysporum* using endophytic or rhizospheric bacterial isolates of hot pepper (*C*. *annum*), but the documented information on biocontrol of this pathogen using *Trichoderma* fungi is very limited. This necessitates the current research to be conducted. Therefore, the present study was focused on the isolation, characterization, and selection of *Trichoderma* strains that have the ability to suppress and limit the growth of *F*. *oxysporum* which causes heavy loss of hot pepper yield in Bure Woreda, Ethiopia.

## 2. Materials and Methods

### 2.1. Description of the Study Area

Hot pepper plants with rhizospheric soil were collected from three selected growing areas of Bure Woreda, West-Gojjam Zone of Amhara National Regional State of Ethiopia. The Woreda is found 160 km southwest of the regional capital city of Bahir Dar and 400 km northwest of Addis Ababa, the capital city of Ethiopia. The Woreda is well known for producing hot peppers at a regional and national level. Three soil types, namely, humic nitosols (63%) found in the wet dega agro-ecology, eutric cambisols (20%) found in the wet woina-dega, and eutric vertisols (17%) are found in the wet and moist lowlands of this Woreda. Bure Woreda is located at 10^o^42′ N 37^o^ 4′E with an elevation ranging from 713 to 2,604 meters above sea level. The average minimum and maximum annual temperature and rainfall of the Woreda are between 14°C and 24°C and 1386 mm to 1757 mm, respectively [10].

### 2.2. Samples and Pathogen Collection

Soil samples for the isolation of antagonists were collected from the rhizosphere of hot pepper. The samples were collected from three selected districts of Bure Woreda that have been under hot pepper cultivation for the past several years. Soil samples were carefully collected and placed in separate clean polyethylene plastic bags before being transported to Debre Markos University via icebox and kept in the refrigerator at 4°C for 48 hours until laboratory analysis was performed. The wilt pathogen *Fusarium oxysporum f.sp.capsici* was obtained from Addis Ababa University. The pathogen was confirmed by growing on Malachite green agar (a selective medium for *Fusarium* spp.) and showing cottony white to pink mycelia with rapid growth (72–96 h).

### 2.3. Isolation and Identification of *Trichoderma* Isolates

A ten-gram sieved soil sample was mixed separately in ninety (90) mL of distilled water in a 150 mL conical flask. The flask was shaken on an orbital shaker for 15 minutes at 240 rpm. Then, serial dilutions (10^−1^, 10^−2^, 10^−3^, 10^−4^, 10^−5^, 10^−6^, and 10^−7^) were made. 0.1 mL suspensions of 10^−4^ to 10^−7^ dilutions were transferred to potato dextrose agar (PDA) (HiMedia, India), spread evenly, and incubated at 28 ± 2°C for 3–7 days. Based on colony morphology, distinct colonies were isolated and purified separately on PDA. The seven-day-old pure fungal isolates were identified according to a taxonomic key for the genus *Trichoderma* using macroscopic and microscopic evaluations such as the mode of mycelia growth; lower and upper colony color; colony texture; pigment secreted into the agar, conidia shape and size; loosely or compactly tufted mycelia; and the formation of distinct concentric rings. Then, the isolates were stored in a refrigerator at 4°C for stress tolerance and antagonistic potential tests. To prevent unwanted bacterial contaminants, the medium was amended with 20 µg/mL of nalidixic acid [11,12].

### 2.4. Dual Culture Test for Antagonistic Fungal Isolates

The antagonist activities of the *Trichoderma* isolates were examined against the pathogen *F*. *oxysporum* using a dual culture technique. The mycelia discs (5 mm, using cork borer) of a 7-day-old pure culture of *F*. *oxysporum f.sp.capsici* were placed on the PDA plates 10 mm away from the edge, and the same size mycelia disc of *Trichoderma* isolate was placed on the opposite edge of the petri dish, whereas control plates were inoculated with only the pathogen disc and incubated at 25 ± 2°C for 7 days. The colony diameter of radial growth of the targeted fungal pathogen *F*. *oxysporum* was measured after the 1^st^, 2^nd^, 3^rd^, and 4^th^ days of incubation at two locations from the center of the test plate, and an average diameter was calculated. Finally, the percent inhibitions of average radial growth were calculated by using the following formula in relation to the growth of the controls [13].

(1) $$\begin{array}{c} L=\left(\frac{C-T}{C}\right)\times 100, \end{array}$$

where *L* = inhibition percentage, *C* = radial growth measurement of the control (without *Trichoderma*) in mm, and *T* = radial growth of the pathogen in the presence of *Trichoderma* species in mm.

### 2.5. Characterization of *Trichoderma* Antagonists for Stress Tolerance

To determine their biocontrol ability at different levels of stress, antagonistically effective isolates were chosen for their *in vitro* physiological resistance capacities such as tolerance to different pH (2, 4, 6, 8, 10, and 12), temperature (5, 10, 15, 35, 40, and 45°C), and salt concentrations (1, 2, 3, 4, 5, 6, 7, and 8%). Finally, all plates were incubated at 28 ± 2°C for 3–5 days [14].

The *Trichoderma* isolates were also tested for their intrinsic resistance to heavy metals, namely chromium (Cr), mercury (Hg), nickel (Ni), zinc (Zn), and lead (Pb) by the agar dilution method. The heavy metals were separately incorporated into Sabouraud dextrose agar (HiMedia, India) at a concentration of 100 ppm. The culture was inoculated and incubated at 28 ± 2°C for 3–5 days, the plates were observed for fungal mycelial growth. To examine the production of hydrolytic enzymes by *Trichoderma* strains, the medium was supplemented with amylase, protease, cellulase, and chitinase enzymes and incubated at 28 ± 2°C for 3–5 days. The clear zone formed surrounding the colony was considered positive for all tests [15].

### 2.6. Data Analysis

The experiments were carried out in triplicate, and the average data was used for each calculation. The results of the *in vitro* antagonistic efficiency test were analyzed and interpreted using one-way ANOVA. The experimental treatments were compared and contrasted against their controls following Duncan's multiple range tests using SPSS version 25 at a significance level of *p* < 0.05.

## 3. Results

### 3.1. *Trichoderma* Strain Characterization and Stress Tolerance Testing

**Figure 1:**
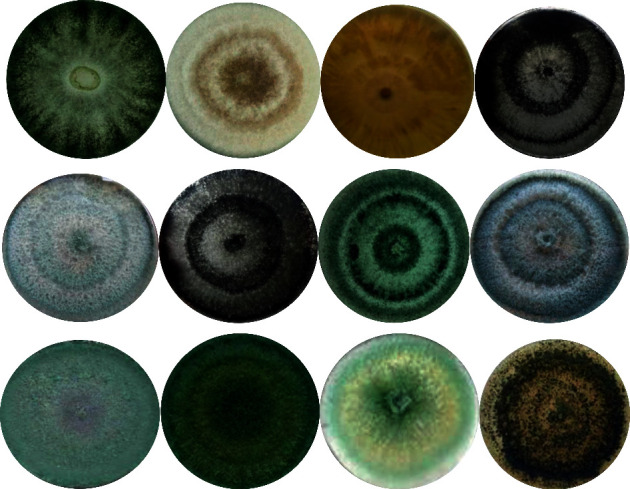
Colony morphology of representative *Trichoderma* isolates grown on PDA.

**Figure 2:**
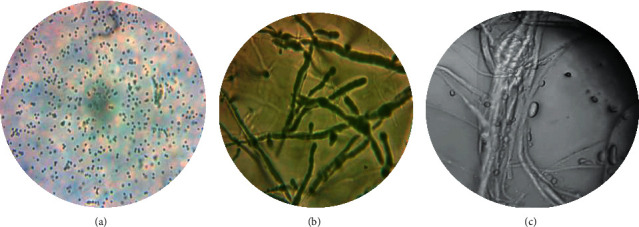
(a) *T*. *viride* spore; (b) *T. viride* mycelia; (c) mass winding and covering effect of *T*. *viride* strain against *F*. *oxysporum*.

A total of 32 *Trichoderma* fungi were isolated from the rhizospheric soil of hot peppers (Figure 1). Based on morphological (colony color, mycelia growth pattern, colony texture, conidia shape, and size) and physiological (growth at different temperatures, pH, NaCl concentration, and tolerance to heavy metals) characteristics, potent isolates (TD1, TD5, TD7, TD11, TD15, and TD21) were tentatively identified as *T*. *harzianum*, *T*. *asperellum*, T. *viride* (Figure 2), *T*. *hamatum*, *T*. *virens,* and *T*. *longibrachiatum*. Among the *Trichoderma* strains, 3 (50.0%), 2 (33.3%), and 1 (16.7%) were dark green, white, and green-white, respectively. Regarding mycelia growth, 5 (83.3%) and 1 (16.7%) of the isolates were raised and flat, respectively (Table 1). Similarly, the isolates showed variation in colony color and reverse color (Figure 1). Concerning microscopic observation, the isolates were morphologically indistinguishable.

All *Trichoderma* isolates showed growth between 10 and 40°C temperature intervals, whereas none of the isolates were grown at 5, 45, or 50°C. An increase in the growth of the isolates was observed as the incubation temperature increased from 15 to 28°C. The *Trichoderma* strains were grown at pH ranges of 4–10 and showed variations in their growth. The maximum number of isolates showed high growth at pH = 4–6 and a minimum at 10. Among the isolates, TD1, TD5, and TD7 showed higher growth than any other isolate at all pH levels. The highest growth of the isolates was recorded at a pH of 6, and they showed a decrease when pH went towards basic (alkaline condition). Concerning salinity tolerance, all of the isolates (100%) were grown at a salinity concentration range of 1–5%. Among the isolates, 4 (66.7%) and 3 (50.0%) were grown at salinity concentrations of 6% and 7%, respectively. There was no isolate showing growth at 8% NaCl concentration (Table 2).

All *Trichoderma* isolates showed positive tolerance for lead and zinc heavy metals. Among the six isolates, five (77.7%), four (88.8%), and two (22.3%) isolates were resistant to nickel, mercury, and chromium, respectively (Table 2).

With regard to the hydrolytic enzyme production (the mycoparasitic activity), variations were observed among *Trichoderma* isolates (Table 3 and Figure 2(c)). Among the isolates, 5 (83.3%) of them were positive for protease, chitinase, and cellulase production, whereas 1 (16.7%) of the isolates was positive for amylase production.

### 3.2. In Vitro Antagonistic Activity of Isolates via Dual Culture Assay

**Figure 3:**
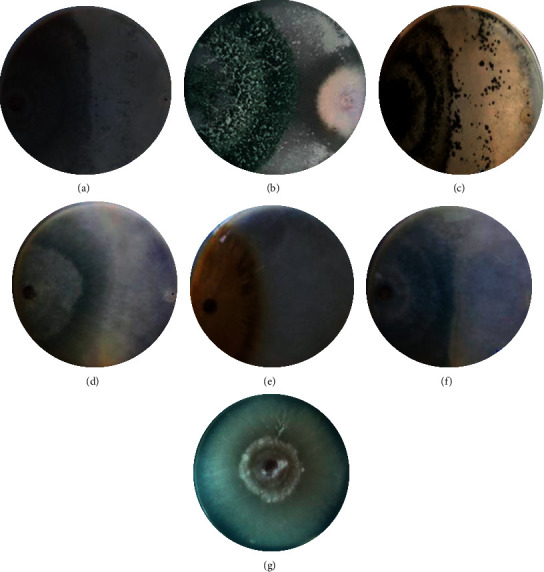
Dual culture assay of *Trichoderma* strains against a fungal pathogen. (a) *T*. *harzianum* + *F*. *oxysporum*, (b) *T*. *asperellum* + *F*. *oxysporum*, (c) *T*. *viride* + *F*. *oxysporum*, (d) *T*. *hamatum* + *F*. *oxysporum*, (e) *T*. *virens* + *F*. *oxysporum*, (f) *T*. *longibrachiatum* + *F*. *oxysporum*, and (g) control (*F*. *oxysporum f.sp.capsici*).

From a total of 32 pure isolates, only 6 isolates inhibited the growth of the pathogen in the dual culture assay and were selected for physiological and biochemical characterization. The antagonistic isolates showed different inhibition efficiencies (Table 3 and Figure 3). Among the 6 isolates tested for dual culture, 4 (66.7%) isolates significantly inhibited the radial growth of the pathogen (>50%), while 2 (33.3%) of the isolates showed less inhibition of radial growth of the pathogen (< 50%). The highest antagonistic efficiency was 93.57% (TD7), followed by 90.12% (TD1) and 89.45% (TD5), while the lowest inhibition efficiency was 48.68% and 45.72% in TD11 and TD15 strains, respectively. Generally, 66.7% of the isolates showed better (> 50%) inhibition of radial growth than the hot pepper pathogen, *F. oxysporum f.sp.capsici* mycelia.

## 4. Discussion

Plant disease needs to be controlled to keep the quality and enhance the productivity of the yields. One of the main constraints that contribute to the low quality and productivity of hot peppers in Ethiopia is failure due to wilt disease caused by *Fusarium oxysporum*. The management of this pathogen through several fungicides results in the accumulation of harmful chemical residues in the soil, water, and grains. The improper utilization (spraying huge quantities and successive dosage units) of fungicides also accelerates the development of fungicide-resistant strains of the pathogen with perilous effects on human as well as animal health and thus leads to ecological imbalances. To alleviate the effect of chemicals (e.g., fungicides), it is important to develop economically safe, long-lasting, and effective biocontrol methods for the control of plant diseases. *Trichoderma* species are one excellent model that has been used as effective soil biocontrol agents with significant antagonistic potential against a wide range of fungal phytopathogens [16,17].

In the present study, from a total of 32 isolates, only 6 *Trichoderma* strains displayed considerable antagonist against radial growth of the hot pepper wilt pathogen (*Fusarium oxysporum f.sp.capsici*). During the dual culture assay, the maximum antagonistic efficiency recorded was 93.57% from the TD7 strain, followed by 90.12% by TD1, and 89.45% by TD5. This finding is consistent with those of Naher et al. [17], who isolated 16 *Trichoderma* isolates, of which *T*. *parareesei* strain TPE7, *T*. *parareesei* strain TPE6, and *T*. *harzianum* strain THLB4 demonstrated strong antagonistic activity with 91%, 89%, and 76% efficiency against the hot pepper pathogen *F*. *oxysporum*, respectively. Ragab et al. [18] had previously reported that URGI 75% WP, Nativo SC 300, and Twinstar75 WG *Trichoderma* strains, respectively, showed 98.8%, 94.0%, and 92.3%, which is comparatively higher mycelial growth inhibition against the fungal pathogen. This might be due to the variations in volatile and non-volatile metabolites produced by *Trichoderma* strains and differences in mechanisms of action against the growth of pathogenic fungi. Nonetheless, Amin and his colleagues [19] reported that the highest percent of inhibition against *F*. *oxysporum* causing chilli wilt was 41.88% observed from the *T*. *viride* (Tv-1) strain, followed by *T*. *viride* (Tv-2) and *T*. *harzianum* (Th-1) with 35.36 and 30.07 percent of inhibition against the radial growth of the test pathogen, respectively. This is lower and inconsistent with the current findings. This might be due to the genes of *F*. *oxysporum* that might be responsible for virulence being highly variable, in which case it breaks the resistance of most pepper varieties.

According to Ibarra-Medina et al. [20], antagonists with over 70% inhibition of pathogen mycelial growth are considered effective biocontrol. Based on this, among the six *Trichodermal* isolates, 3 (50.0%) of the isolates were considered effective antagonists. In contrast, Kannangara and Dharmarathna [21] described the *Trichoderma* antagonists with inhibition efficiency higher than 40% as a better biological control agent. According to this description, all the isolates indicate higher inhibition (> 40%) efficiency; therefore, all the isolates can be considered as better biocontrol control agents.

With regard to the hydrolytic enzyme production (the mycoparasitic activity), variations were observed among *Trichoderma* isolates. Microscopic examination of the interaction between *Trichoderma* and pathogens revealed that *Trichoderma* has a strong antagonistic potential against the fungal pathogen *Fusarium oxysporum f*.*sp*.*capsici* via mycoparsitism (coiling, penetration, direct contact, and parallel growth alongside the host hyphae). As reported by Brimner and Boland [22], antagonists have the ability to produce enzymes that catalyze the breakdown of the fungal cell wall, which is consistent with the present finding. Sharma et al. [23] also studied the lytic enzymes of *Trichoderma* such as chitinases, glucanases, and proteases that degrade the host cell wall and kill the pathogen. This might be due to *Trichoderma* being good producers of chitinases that hydrolyze the glycosidic bonds between the N-acetyl glucosamine residues of chitin. This genus also releases cellulases which hydrolyze *β*-1, 4 glucans cell wall component of the phytopathogenic fungi. Furthermore, the idea is supported by Agrawal and Kotasthane [24].

In the current study, antagonistically effective isolates were subjected to different degrees of physiological stress (temperature, pH, salt concentrations, and heavy metals) to determine their stress tolerance, as well as biocontrol ability at different conditions. Several reports have indicated that the biocontrol efficiency of *Trichoderma* may differ in regions due to various agro-climatic conditions [25]. The growth increment of the isolates from 15 to 28 ± 2°C was in parallel with the report by Harrison et al. [26]. *Trichoderma* isolates' growth rates were increased when the temperature increased from 20 to 30°C. Similar to the present finding, Ragab et al. [18] and Daryaei et al. [27] also reported that none of the *Trichoderma* isolates grew at 5°C and above 40°C. This could be because high temperatures inhibit *Trichoderma* growth and conidium production while low temperatures inhibit conidium production and germination [28]. High temperatures also cause cell contents such as carbohydrates, lipids, and proteins to degrade and clump [29]. In this study, the influence of pH on the mycelial growth of *Trichoderma* clearly indicates that an acidic pH (4–6) is preferable than a basic pH. Consistent results reported by Jackson et al. [30] showed that a *T*. *harzianum* isolate showed optimum mycelial growth between pH 4.8 and 6.8. The better growth of *Trichoderma* in acidic conditions is also supported by other studies conducted elsewhere by Edward and Desalegne [31], acidic pH favored fungal growth more than alkaline conditions. This implies the increased pH level of soil may inhibit the growth of *F*. *oxysporum* in soil too. With regard to salinity tolerance, all of the 6 (100%) *Trichoderma* isolates were grown at a range of 1–5% salinity concentration, and they were inhibited when going toward more halophilic conditions. All isolates were resistant to lead and zinc, while variations were observed against other heavy metals. As a result of their ability to grow under a variety of physiological stresses, the isolates proved to be effective antagonists for the biocontrol of the *Fusarium oxysporum f*.*sp*.*capsici* pathogen of hot pepper.

## 5. Conclusions

The results of this study revealed that out of 32 *Trichoderma* isolates tested *in vitro*, six (*T*. *harzianum* (TD1), *T*. *asperellum* (TD5), *T*. *viride* (TD7), *T*. *hamatum* (TD11), *T*. *virens* (TD15), and *T*. *longibrachiatum* (TD21)) strains showed varying (45.72–93.57%) antagonistic activities towards the tested pathogen. Of six isolates, 3 (TD5, TD1, and TD7) strains showed the most effective antagonists against the root pathogen *Fusarium oxysporum f.sp.capsici* of hot pepper (C. *annum* L.) with a colonization percentage of 89.45%, 90.12%, and 93.57%, respectively. These *Trichoderma* strains showed higher inhibition efficiency (>70%) against the mycelial growth of *F*. *oxysporum* across a wide range of physiological tolerance (temperature, pH, salt concentration, and heavy metals). The isolates also produce different hydrolytic enzymes (amylase, protease, cellulase, and chitinase) with mycoparasitism potential against the mycelia growth of *F*. *oxysporum f.sp.capsici*. Thus, the results of this study concluded that TD1, TD5, and TD7 *Trichoderma* strains have become candidates for developing a biocontrol agent against the root pathogen *F*. *oxysporum f.sp.capsici* of hot pepper.

## Figures and Tables

**Table 1:** Detailed macro- and microcharacteristics of *Trichoderma* strains isolated from hot pepper rhizospheric soil.

Strains	Colony color	Reverse color	Mycelia growth	Colony texture	Conidia shape	Conidia size (*μ*m)	Tentative identification
TD1	Dark green	Bright green	Raised	Floccose	Subglobose	3.1–3.7 × 4.1–4.5	*Trichoderma harzianum*
TD5	Dark green	Dark green	Raised	Floccose	Globose to obovoid	4.1–4.4 × 3.0–3.5	*Trichoderma asperellum*
TD7	Dark green	Pale green	Raised	Arachnoid	Subglobose	2.6–3.1 × 2.0–2.9	*Trichoderma viride*
TD11	Green	White	Raised	Floccose	Obovoid to ellipsoidal	3.1–3.5 × 4.1–4.5	*Trichoderma hamatum*
TD15	Yellow	White	Flat	Arachnoid	Subglobose to ellipsoid	4.0–4.8 × 3.5–4.0	*Trichoderma virens*
TD21	Green white	Yellow	Raised	Floccose	Ellipsoidal	4.2–5.9 × 2.9–4.5	*Trichoderma longibrachiatum*

**Table 2:** Physiological stress tolerance testing of six potent *Trichoderma* strains under various conditions.

Strains/characteristics	Growth at different temperatures in °C							Growth at different pH						Growth at different NaCl concentrations								Tolerance to heavy metals				
	5	10	15	35	40	45	50	2	4	6	8	10	12	1	2	3	4	5	6	7	8	Cr	Hg	Ni	Zn	Pb
TD1	−	+	+	+	+	−	−	+	+	+	+	+	−	+	+	+	+	+	−	+	−	−	+	−	+	+
TD5	−	+	+	+	+	−	−	+	+	+	+	−	−	+	+	+	+	+	−	+	−	+	+	+	+	+
TD7	−	+	+	+	+	−	−	+	+	+	+	+	−	+	+	+	+	+	+	+	−	+	+	+	+	+
TD11	−	+	+	+	−	−	−	+	+	+	+	+	−	+	+	+	+	+	+	−	−	−	−	+	+	+
TD15	−	+	+	+	+	−	−	+	+	+	+	−	−	+	+	+	+	+	−	−	−	−	+	+	+	+
TD21	−	+	+	+	−	−	−	+	+	+	+	−	−	+	+	+	+	+	+	−	−	−	−	+	+	+

+ = positive, − = negative, Cr= chromium, Hg = mercury, Ni= nickel, Zn = zinc, and Pb = lead.

**Table 3:** Hyperparasitism mechanism of *Trichoderma* strains via hydrolytic enzymes and their *in vitro* antagonistic activity via antibiotic production.

Strains code	Hydrolytic enzymes				Mean of triplicate dual culture inhibition (mm)	% of antagonistic efficiency of *Trichoderma* against *F*. *oxysporum*
	Amylase	Protease	Chitinase	Cellulase		
TD1	−	+	+	+	63 ± 0.21^ab^	90.12
TD5	−	+	+	+	61 ± 0.15^ef^	89.45
TD7	+	+	+	+	66 ± 0.13^ba^	93.57
TD11	−	+	−	+	34 ± 0.32^hg^	48.68
TD15	−	−	+	+	32 ± 0.14^gh^	45.72
TD21	−	+	+	−	43 ± 0.67^cb^	65.84

+ = positive; − = negative.

## Data Availability

The datasets used to support the findings of this study are included within the article.
